# Adipose transcriptome in the scalp of androgenetic alopecia

**DOI:** 10.3389/fmed.2023.1195656

**Published:** 2023-09-07

**Authors:** Criselda Jean G. Cruz, Yi-Kai Hong, Wilson Jr. F. Aala, Ren-Yeu Tsai, Pei-Lun Chung, Yau-Sheng Tsai, Chao-Kai Hsu, Chao-Chun Yang

**Affiliations:** ^1^Department of Dermatology, National Cheng Kung University Hospital, College of Medicine, National Cheng Kung University, Tainan, Taiwan; ^2^International Center for Wound Repair and Regeneration, National Cheng Kung University, Tainan, Taiwan; ^3^Institute of Clinical Medicine, College of Medicine, National Cheng Kung University, Tainan, Taiwan; ^4^Department of Dermatology and Skin Laser Center, Taipei Municipal Wan-Fang Hospital, Taipei Medical University, Taipei, Taiwan

**Keywords:** androgenetic alopecia, hair follicle, adipogenesis, PPAR signaling pathway, transcriptome

## Abstract

Previous studies have shown how adipocytes can modulate the activity of hair follicle stem cells. However, the role of adipocytes in the pathogenesis of androgenetic alopecia (AGA) remains unknown. We aimed to determine signaling pathways related to the adipose tissue changes in the human scalp with AGA through RNA-seq analysis. RNA was isolated from the adipose tissues derived from the bald (frontal) and normal (occipital) scalps of male patients with AGA (*n* = 4). The pooled RNA extracts from these samples were subjected to RNA sequencing, followed by differential gene expression and pathway analysis. Our gene expression analysis identified 1,060 differentially expressed genes, including 522 upregulated and 538 downregulated genes in the bald AGA scalp. Biological pathways pertaining to either adipose tissue metabolism or the hair cycle were generated in our pathway analysis. Downregulation of the peroxisome proliferator-activated receptor (PPAR) signaling pathway was noted to be significant in the bald scalp. Expression of adipogenic markers (e.g., *PPARG, FABP4, PLN1*, and *ADIPOQ*) was also decreased in the bald site. These findings imply that adipogenesis becomes downregulated in AGA, specifically within the bald scalp adipose. Our results lead to the hypothesis that PPARγ-mediated adipogenesis in the scalp adipose, *via* crosstalk with signaling pathways involved in hair cycling, might play a role in AGA.

## 1. Introduction

Androgenetic alopecia (AGA) is a highly prevalent hair loss disorder affecting both men and women globally. Genetic predisposition and androgen sensitivity are among the few factors identified in the progression of AGA. The majority of the studies attempting to unravel the mechanisms behind AGA mainly focus on the hair follicle (HF), yet there is evidence that adipocytes themselves can regulate hair follicle stem cell (HFSC) activation through the expression of bone morphogenetic protein and platelet-derived growth factor (PDGF) (1, 2). Moreover, there was a significant reduction in the scalp adipose thickness in the bald scalp of male AGA patients vs. the normal scalp (3, 4). It has been previously reported that the dermal adipose layer changes its thickness throughout the hair cycle (5). During anagen, the adipose tissue proliferates as the HF grows downward, allowing the hair bulb to become adjacent to dermal adipocytes. As the HF enters telogen, the thickness of the underlying adipose tissue notably decreases. This implies that proliferation of the scalp adipose tissue is presumably halted or minimally occurring in AGA, wherein the HF are either arrested in telogen or miniaturized in anagen. In effect, the signaling and metabolic processes involving both the HF and adipose tissue are then likely altered in the bald AGA scalp. Given all these findings, it is plausible that the scalp adipose tissue and its metabolism participate in the pathogenesis of AGA.

## 2. Methods

**Figure 1:**
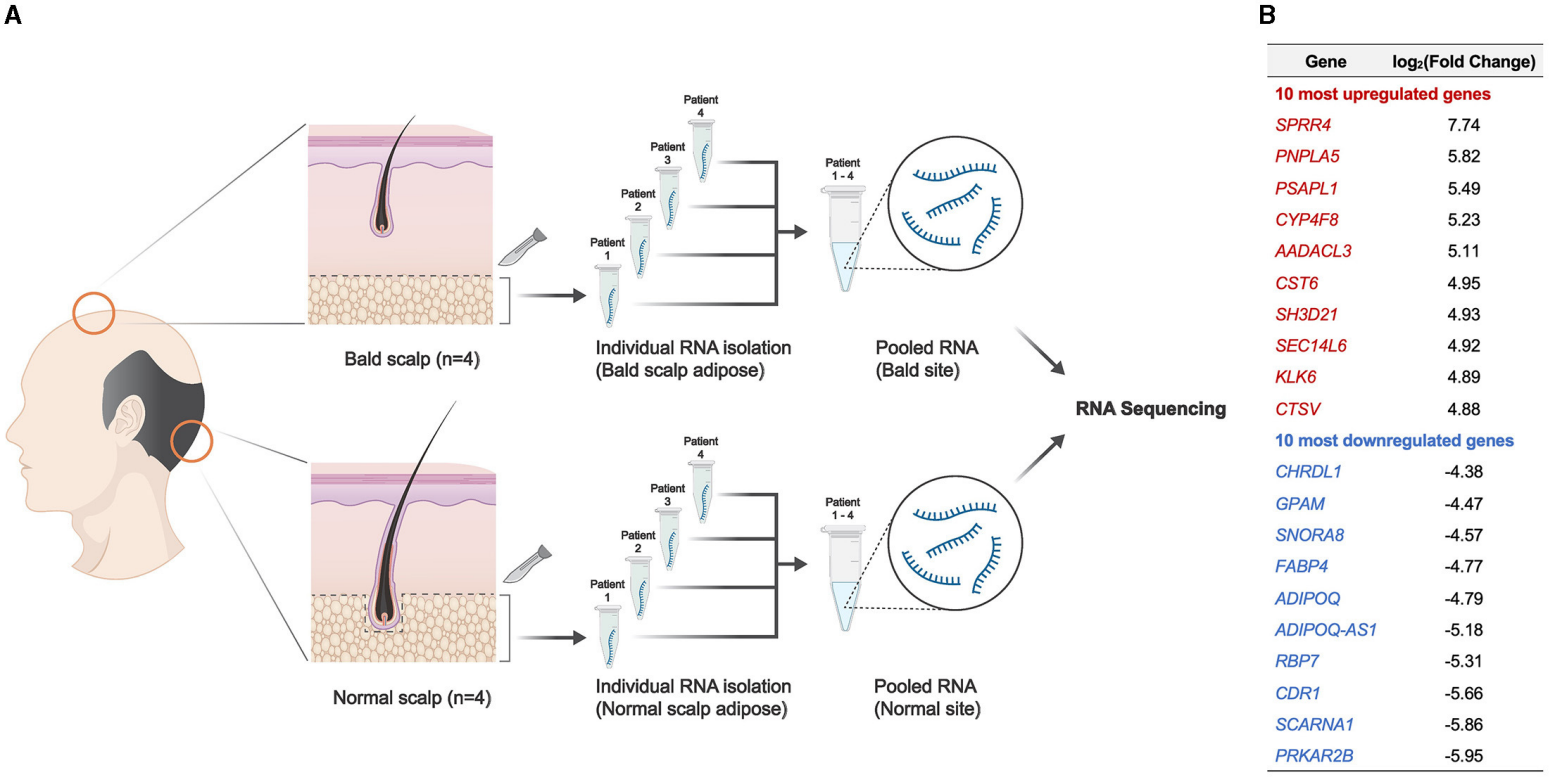
Overview of the experimental design and resulting DEGs from adipose tissue in the scalp. **(A)** Schematic representation of the experimental design. **(B)** Ten most upregulated and downregulated DEGs (*p* < 0.05) resulting from RNA sequencing analysis.

In this study, we aimed to identify candidate signaling pathways that are likely to be affected by AGA within the scalp adipose layer by utilizing RNA-seq analysis tools. Ethical approval was obtained from the Institutional Review Board of National Cheng Kung University Hospital. Participant characteristics are summarized in Table 1. Adipose tissue samples from male participants with AGA (*n* = 4) were collected through punch biopsy at two different sites: bald (frontal) and normal (occipital, as control) scalps (Figure 1A). The epidermis and dermis, including the HF, were removed under a dissecting microscope prior to RNA extraction of the remaining adipose tissue. RNA isolates were obtained following the standard TRIzol^®^ reagent protocol (Invitrogen, USA) for RNA extraction, pooled (*n* = 4 per collection site) for sufficient input RNA material, and then sent to the National Cheng Kung University Center of Genomic Medicine (Tainan, Taiwan) for RNA sequencing and gene expression analysis. The whole transcriptome library was prepared using the SMARTer^®^ Universal Low Input RNA Kit (Takara Bio, USA) and the 5500 SOLiD^TM^ Fragment Library Core Kit (Applied Biosystems, USA) and then subjected to sequencing with the 5500xl SOLiD^TM^ Sequencer (Applied Biosystems, USA). The dataset utilized in this study is available at NCBI's Gene Expression Omnibus database (GEO ID: GSE212757). Normalized counts and the corresponding fold change were generated using Partek software.

**Table 1:** Participant characteristics with values presented as median (range) or mean ± standard deviation.

**Characteristics**	**Value**
Men (n)	4
Age (years)	39 (36–51)
Body weight (kg)	72.3 ± 13.4
Height (cm)	167.3 ± 2.2
Body mass index (kg/m^2^)	25.7 ± 4.1
AGA Grade (Norwood–Hamilton classification)	V–VII

## 3. Results

**Figure 2:**
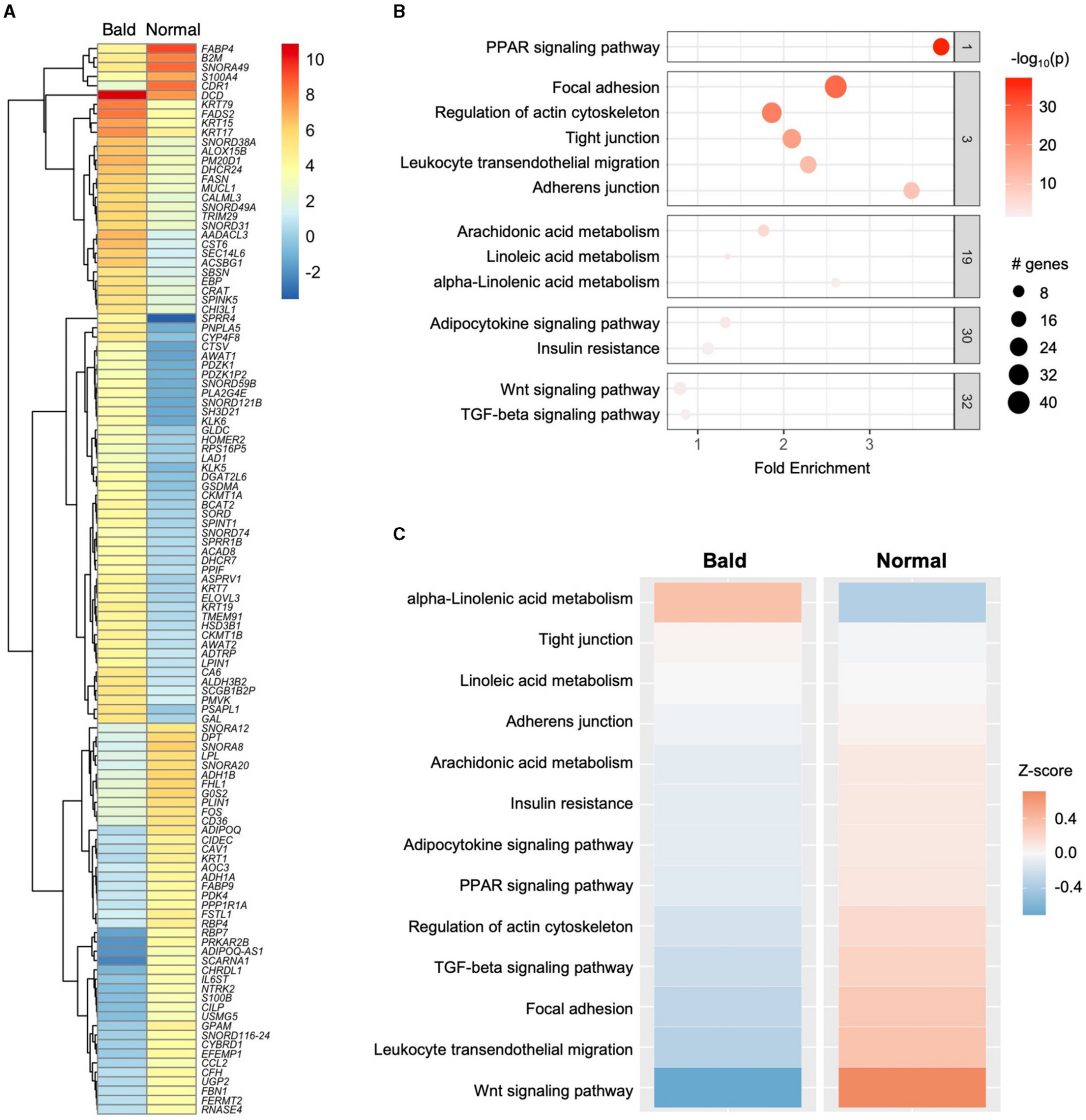
Transcriptomic profile of the scalp adipose in AGA. **(A)** Heatmap of identified DEGs with |log_2_FC| > 3 and *p* < 0.05. **(B)** Clustered terms obtained from pathway enrichment analysis using pathfindR. **(C)** Enriched pathways arranged according to Z-score (upregulated–orange; downregulated–blue).

A total of 1,060 differentially expressed genes (DEGs) were identified (|log2(fold change)| ≥ 1 and adjusted *p* < 0.05), with 10 of the most upregulated and downregulated significant DEGs (ranked according to fold change), as shown in Figure 1B. Among the 1,060 DEGs, there were 522 upregulated genes and 538 downregulated genes in the bald scalp adipose. Transcript expression values (RPKM) of the top 100 upregulated and downregulated genes are indicated in Supplementary Table S1. A heatmap was generated to illustrate DEGs exhibiting |log2(fold change)| > 3 and an adjusted *p*-value of < 0.05 (Figure 2A). Identified DEGs were subjected to further analysis using pathfindR (version 1.6.3) with the Kyoto Encyclopedia of Genes and Genomes as the gene set. Pathway enrichment analysis of our dataset revealed several processes relevant to adipose tissue metabolism, i.e., fatty acid metabolism, adipocytokine signaling pathway, and insulin resistance, as well as those involved in the hair cycle, namely Wnt signaling and TGF-beta signaling pathway (Figures 2B, C). It is worth noting that the peroxisome proliferator-activated receptor (PPAR) signaling pathway is enriched and significantly downregulated in the bald AGA scalp. Among the identified DEGs in the PPAR signaling pathway, an adipogenic gene, *PPARG*, is significantly downregulated in the bald frontal scalp group with a fold change of −2.94 (*p* < 0.05).

## 4. Discussion

Our transcriptomic analysis of adipose tissue retrieved from the bald scalp of AGA patients revealed that, among the identified DEGs, several adipogenic genes, e.g., *PPARG, FABP4, PLN1*, and *ADIPOQ*, and the PPAR signaling pathway were all found to be downregulated; hence, this study emerges to be in congruence with previous reports that empirically tackled the influence of adipocyte-derived transcription factors and proteins involved in adipogenesis on HF (1, 6–15). Adipogenesis refers to the maturation of adipocyte precursor cells into mature adipocytes and a well-studied regulator of which is PPARγ (*PPARG*) (16). Having known this, results of our study thereby suggest that adipogenesis via PPARγ is likely to be downregulated in the bald scalp of AGA patients.

*PPARG* expression was reported to be remarkably decreased in the scalp tissue of lichen planus pilaris (LPP) patients compared to healthy controls, as quantified by real-time polymerase chain reaction (9). Furthermore, Karnik et al. (9) performed a functional study using human keratinocytes and outer root sheath cells *in vitro*, which revealed that treating these cells with PPARγ-specific agonists, e.g., ciglitazone, rosiglitazone, pioglitazone, and troglitazone, resulted in the modulation of the proinflammatory lipid metabolism and peroxisome biogenesis, which further implies that PPARγ may have a direct influence on human pilosebaceous units.

Activation of PPARγ signaling also upregulated the expression of keratin 15 and 19 in *ex vivo* human HF, demonstrating its beneficial role in promoting the survival of HF progenitor cells (11, 12). As a form of loss-of-function validation, knocking out *PPARG* specifically in mouse HFSC resulted in progressive hair loss upon reaching 3 months of age, despite being previously normal at birth (9). In addition, histopathology of skin specimens from these mice showed follicular scarring and permanent hair loss, resembling the features of LPP in humans. Sardella et al. (13) further demonstrated a transient delay in HF morphogenesis, decreased expression of the relevant differentiation markers and factors, and subsequent scarring of hair follicles in PPARγ-null mice. Taken together, these data suggest that PPARγ could be contributing significantly to both normal HF physiology and pathological conditions.

Apart from PPARγ, the downregulation of adiponectin is also worth noting in our dataset as more studies emerge regarding this adipose-derived protein in relation to hair loss. Adiponectin receptors were documented to be present in human HF, particularly along the outer root sheath and dermal sheath, as confirmed by immunofluorescent studies (14). As for its hair-related function, adiponectin appeared to have hair growth-promoting activity when added to human HF maintained *ex vivo*, exhibiting a notable increase in hair shaft length, i.e., 1.0 mm for adiponectin-treated HF compared to 0.8 mm for minoxidil-treated HF after 6 days of treatment. Moreover, this visibly quantifiable effect of adiponectin on HF was reported to be dose-dependent, with remarkable augmentation of proliferative epithelial cells, as demonstrated by Ki67 immunostaining. A recent study by Ohn et al. (10) also reported similar results in terms of the hair growth-promoting effect of adiponectin and an adiponectin-derived pentameric peptide (P5), which was designed for transdermal delivery.

Leptin, another adipocyte-related protein, also potentially contributes to hair regeneration, as documented in previous studies. For instance, expression of leptin receptors (LEPR) in the dermal papilla (DP) and along the outer bulge epithelial layer of both mouse and human HF is highly suggestive that leptin might, in fact, have a direct impact on HF growth (7, 15). In addition, the mixture of LEPR^+^ DP cells (after sorting by flow cytometry) and epithelial stem cells was shown to be successful in producing hair on the skin of nude mice, thereby further demonstrating that the presence of leptin might indeed be in favor of HF regeneration (7). While these investigations on the commonly known adipogenic genes and proteins remain ongoing, some studies have opted to explore specific cell types within the adipose tissue. One example would be skin-derived adipocyte precursor cells, especially since these cells were found to highly express platelet-derived growth factor A (PDGFA), which mediates HF cycling as demonstrated by loss-of-function genetic tools in animal studies, i.e., *PDGFA*-null mice presented with abnormally thinner hair with smaller DP compared to that of the wild-type controls, hence suggesting a lack and/or delay in the activation of HFSCs (1, 8).

With elevated dihydrotestosterone (DHT) near the DP in AGA, downregulation of Wnt signaling occurs through increased expression of the potent Wnt inhibitor, dickkopf-1 (DKK-1) (17). One possible sequela of Wnt signaling inhibition would be decreased production of transient-amplifying cells (TAC) in the HF epithelium. These TAC may play a crucial, albeit indirect, role in the changes within the adipose tissue during anagen. Aside from promoting HFSC self-renewal and HF downgrowth, sonic hedgehog (Shh) from TAC promotes the expression of PPARγ, suggesting that adipogenesis could be activated in this particular phase of the hair cycle (18). However, in AGA, wherein the majority of the HF remains in telogen, failure to initiate anagen could antagonize the degree of HFSC activation and TAC production; hence, a decline in Shh expression from TAC would follow. In effect, and as reflected in our data showing decreased expression of adipogenic markers (e.g., *PPARG, FABP4, PLN1*, and *ADIPOQ*) in the bald scalp, PPARγ-mediated adipogenesis might then be downregulated in the scalp region with AGA.

**Figure 3:**
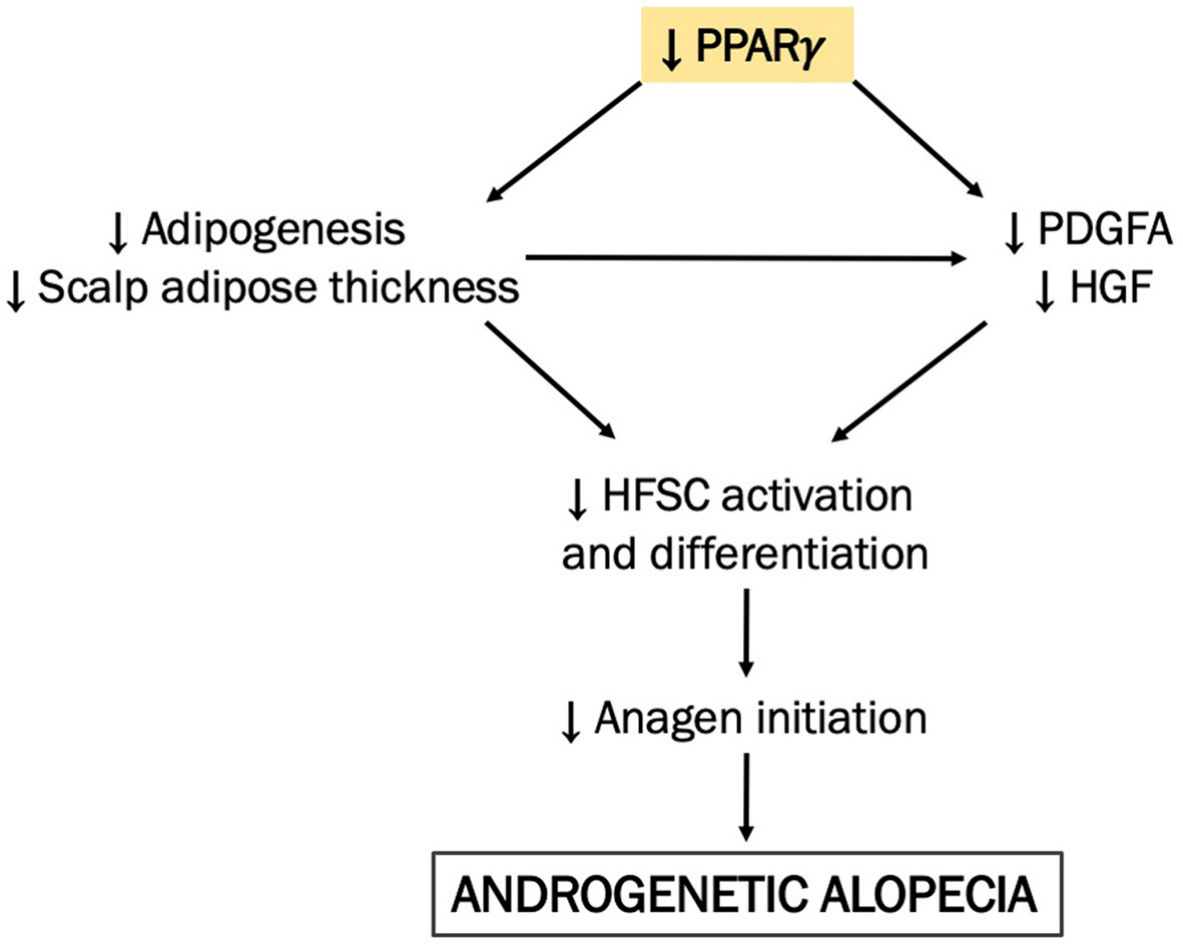
A proposed mechanism integrating scalp adipose metabolism in the pathogenesis of AGA. We hypothesize that adipogenesis in the bald AGA scalp is decreased *via* the downregulation of PPAR signaling *via* PPARγ, resulting in the reduction of the scalp adipose layer thickness. In effect, the resulting gap between the miniaturized HF and scalp adipose becomes even more prominent in the bald scalp, diminishing the corresponding action of adipocyte-derived factors (e.g., HGF and PDGFA) on the HFSC. As an effect, HFSC activation and differentiation are thereby reduced, along with adipogenesis in the scalp adipose tissue, creating an unfavorable environment for hair growth and regeneration.

Changes in adipose tissue thickness potentially influence hair cycle signaling pathways, particularly those that might require the hair bulb and adipocytes to be in close proximity. Preadipocytes can secrete factors, e.g., hepatocyte growth factor (HGF) (19) and PDGFA (1), both of which activate Wnt signaling in the human HF. However, such signaling pathways may be interrupted in AGA if adipogenesis through PPARγ is downregulated, i.e., the adipose layer does not expand from its baseline level as in telogen, thus preventing direct communication between HF and adipocytes. The resulting gap between the HF and adipose in AGA may then prevent adipocyte-derived factors from effectively reaching their binding sites in the HF, thereby downregulating the Wnt signaling pathway (in addition to DKK1-mediated Wnt inhibition) and overall making it unfavorable for HF growth and regeneration. A cascade of events involving PPAR signaling *via* PPARγ in the adipose as one of the mechanisms behind the significant reduction of the scalp adipose in AGA is hereby proposed (Figure 3). Further investigation verifying the mRNA and protein levels with a larger sample size is vital to validate our findings.

Since the adipose layer was manually dissected from the epidermal and dermal layers using a scalpel in this study, the presence of other cell types from the upper skin layers may be inevitable. For instance, *SPRR4*, which is well-known to be enriched in the epithelium (20), has been noted to be upregulated in our dataset, suggesting that cells from the epidermis might have been present in the obtained adipose tissue prior to RNA isolation. Utilizing a laser-capture microdissection technique might help address this issue to better obtain the target tissue or cell population while minimizing the potential contamination of other cell types. Nevertheless, a significant number of key adipose-related genes have been identified to be differentially expressed in our generated dataset, implying that the majority of our retrieved samples comprised RNA from the adipose.

Our study serves as a prelude to characterizing scalp adipose metabolism in AGA through a transcriptomic approach. With the pathophysiology of AGA not yet fully elucidated to date, this study hereby proposes a different perspective, in addition to the HF itself, in deciphering this common hair loss disorder. Future studies to further investigate the molecular and phenotypic alterations of dermal adipocytes in AGA-affected scalps using high-throughput platforms (e.g., single-cell RNA sequencing, high-parameter flow cytometry, and spatial transcriptomics) are mandatory.

## Data availability statement

The datasets presented in this study can be found in online repositories. The names of the repository/repositories and accession number(s) can be found below: Gene Expression Omnibus (accession number GSE212757).

## Ethics statement

The studies involving humans were approved by the Institutional Review Board of the National Cheng Kung University Hospital, Tainan, Taiwan. The studies were conducted in accordance with the local legislation and institutional requirements. The participants provided their written informed consent to participate in this study.

## Author contributions

Conceptualization: CC, C-CY, and C-KH. Funding acquisition: C-CY. Investigation: R-YT and P-LC. Formal analysis and visualization: CC, Y-KH, and WA. Resources: R-YT. Writing—original draft preparation: CC and C-CY. Writing—review and editing: CC, C-CY, Y-ST, and C-KH. All authors contributed to the article and approved the submitted version.
