# Supplementary material for: Adipose transcriptome in the scalp of androgenetic alopecia
Source: Front Med (Lausanne). 2023 Sep 7;10:1195656. doi: 10.3389/fmed.2023.1195656 (PMC10513442; doi:10.3389/fmed.2023.1195656)
Supplement: Supplementary file 1 [file Table_1.DOCX]

**SUPPLEMENTARY MATERIAL**

**Table S1.** Transcript expression values of upregulated (red) and downregulated (blue) genes in the scalp adipose tissue in AGA arranged according to fold change (highest to lowest, p<0.05); values shown as reads per kilobase of transcript per million reads mapped (RPKM).

| **Gene ID** | **Bald (frontal) scalp adipose (pooled samples = 4)** | **Normal (occipital) scalp adipose (pooled samples = 4)** |
| --- | --- | --- |
| *RBAK-RBAKDN* | 4.54607 | 4.83E-07 |
| *SNORD58B* | 4.70535 | 1.01E-06 |
| *PTGES3L-AARSD1* | 4.32037 | 1.17E-06 |
| *SNORA17* | 7.13569 | 6.26E-06 |
| *PRR4* | 17.5228 | 1.88E-05 |
| *TMEM110-MUSTN1* | 26.5352 | 2.94E-05 |
| *PVRIG2P* | 29.2379 | 7.46E-05 |
| *PCNA-AS1* | 11.4207 | 3.68E-05 |
| *MIR1184-1_dup1* | 2.51214 | 1.59E-05 |
| *HSPB2-C11orf52* | 7.8916 | 9.44E-05 |
| *FAM72C_dup2* | 6.99428 | 0.00013055 |
| *KCNJ18* | 5 | 0.00011827 |
| *KLRC4-KLRK1* | 1.90036 | 0.00014725 |
| *FAM231D* | 7.79583 | 0.00152188 |
| *MEF2B* | 2.99919 | 0.00781281 |
| *ANKRD20A2_dup2* | 1.99205 | 0.00614909 |
| *FAM156B_dup2* | 60.1163 | 0.185775 |
| *TCEB3CL2_dup2* | 1.99978 | 0.0137839 |
| *TCEB3C_dup1* | 1.99978 | 0.0137839 |
| *RPL13AP5* | 74.0361 | 0.570671 |
| *SPRR4* | 429 | 4 |
| *MUC19* | 253 | 3 |
| *FAM133DP* | 3.9952 | 0.0520861 |
| *LOC100996758* | 56.9986 | 1.00586 |
| *FAM25C_dup2* | 3.99999 | 0.0709611 |
| *ST20-MTHFS* | 2.02401 | 0.0388715 |
| *LINC00930* | 51 | 1 |
| *MIR6125* | 2.26205 | 0.0448667 |
| *IL12A-AS1* | 278 | 6 |
| *PADI4* | 185 | 4 |
| *LOC649133* | 40 | 1 |
| *CYP4F2* | 439 | 11 |
| *ACSM1* | 113 | 3 |
| *DLX5* | 37 | 1 |
| *LOC101927318* | 30.6768 | 1 |
| *HBG1* | 33 | 1 |
| *ITGA2B* | 32 | 1 |
| *ACY1* | 79.1087 | 2.85238 |
| *SLC26A8* | 29 | 1 |
| *PNPLA5* | 1835 | 64 |
| *CCDC64* | 242 | 9 |
| *SEC14L3* | 51 | 2 |
| *PAPL* | 130 | 5 |
| *EXTL1* | 26 | 1 |
| *ANO7* | 26 | 1 |
| *HELT* | 25 | 1 |
| *KRT9* | 270 | 11 |
| *LINC01164* | 49 | 2 |
| *SLC22A31* | 48 | 2 |
| *EGFL8* | 10.7022 | 0.4563 |
| *CYP2D6* | 20.8145 | 0.985228 |
| *CHRFAM7A* | 23 | 1 |
| *PSAPL1* | 5917.33 | 262.02 |
| *IPO11-LRRC70* | 18.8807 | 0.858029 |
| *ACER1* | 302 | 14 |
| *LOC100288123* | 21 | 1 |
| *KLRG2* | 21 | 1 |
| *FAM207A* | 21 | 1 |
| *C1orf145* | 11 | 1 |
| *MYBPH* | 20 | 1 |
| *ARHGAP40* | 215 | 11 |
| *GLYATL1* | 95 | 5 |
| *ITIH6* | 19 | 1 |
| *CYP4F8* | 1685.93 | 89.0032 |
| *LOC101927322* | 18.5447 | 1 |
| *CLDN10-AS1* | 18.3404 | 1 |
| *LIPH* | 162 | 9 |
| *KRT4* | 288 | 16 |
| *KCNE1L* | 18 | 1 |
| *FAM90A2P* | 18 | 1 |
| *TJP3* | 89.9999 | 5 |
| *AADACL3* | 12476.8 | 718.923 |
| *WFDC2* | 34 | 2 |
| *LOC101927720* | 17 | 1 |
| *DGCR5* | 20 | 1 |
| *ZNF816-ZNF321P* | 21.2248 | 1.2711 |
| *DDX11L5* | 16 | 1 |
| *RAB40A* | 16 | 1 |
| *C16orf74* | 16 | 1 |
| *UBQLN3* | 16 | 1 |
| *PRB3* | 16 | 1 |
| *CPLX3* | 16 | 1 |
| *GAL3ST2* | 16 | 1 |
| *TGM5* | 511.838 | 33 |
| *CST6* | 1783.93 | 115 |
| *MAGEA11* | 31 | 2 |
| *SLC35F4* | 31 | 2 |
| *SH3D21* | 878.844 | 57 |
| *SEC14L6* | 2822.59 | 185 |
| *PGLYRP4* | 30 | 2 |
| *ASIC3* | 15 | 1 |
| *KLK6* | 540.898 | 36 |
| *FOLR1* | 38 | 3 |
| *LOC101928388* | 680.393 | 45.7626 |
| *TG* | 341.158 | 23 |
| *CTSV* | 1483 | 97.0001 |
| *LOC100129316* | 29 | 2 |
| *HBG2* | 29 | 2 |
| *MSH5-SAPCD1* | 32.4334 | 2.25658 |
| *LINC00885* | 268 | 19 |
| *SNORD116-7_dup1* | 0.5 | 25.5 |
| *MFAP5* | 9 | 462 |
| *TNFRSF11B* | 1 | 52 |
| *MUSTN1* | 4.03079 | 209.664 |
| *FABP4* | 533 | 28832.7 |
| *ADIPOQ* | 183.666 | 10083.3 |
| *SNORA13* | 1 | 55 |
| *CPA3* | 3 | 167.64 |
| *MMD* | 12 | 671.419 |
| *RORB* | 2 | 112 |
| *MIR100HG* | 4 | 227.029 |
| *TLR4* | 7 | 409.999 |
| *ZNF285* | 1 | 61 |
| *KLHL31* | 6 | 346.019 |
| *NAP1L5* | 1 | 58 |
| *COL6A5* | 1 | 58 |
| *GIMAP1-GIMAP5* | 1.94176 | 114.73 |
| *GLYAT* | 3 | 149.485 |
| *KCNT2* | 1 | 61 |
| *KCNJ8* | 2 | 121 |
| *FAM47E-STBD1* | 2.56128 | 156.932 |
| *C4orf32* | 5 | 310 |
| *EGFL6* | 23 | 1427 |
| *GNG2* | 16 | 1009 |
| *PDE3B* | 32 | 2041.89 |
| *ICA1L* | 5 | 82 |
| *SYPL1* | 5 | 327.001 |
| *PDGFD* | 10 | 654.062 |
| *CMBL* | 5 | 327 |
| *RPL39* | 1 | 66 |
| *NUP35* | 1 | 73 |
| *PHOSPHO2-KLHL23* | 1.22517 | 86.1096 |
| *ADIPOQ-AS1* | 29.3336 | 2112.61 |
| *CEP57L1* | 1 | 78.2129 |
| *PCOLCE2* | 14 | 1081.21 |
| *TGM3* | 1 | 78 |
| *RBP7* | 7 | 552.599 |
| *CDO1* | 6 | 489 |
| *LOC653653* | 1 | 83 |
| *FHL5* | 4 | 373.001 |
| *GNG11* | 6 | 504 |
| *NDNF* | 1 | 84 |
| *DOCK11* | 22 | 1858.35 |
| *TMEM47* | 4 | 344.161 |
| *ELTD1* | 4 | 346 |
| *MMRN1* | 2 | 187.341 |
| *LEPR* | 13.5071 | 1114.92 |
| *SCHIP1* | 0.431176 | 26.0021 |
| *SGCB* | 7 | 678.056 |
| *GMFG* | 1 | 97 |
| *CDR1* | 245 | 24591.3 |
| *FAM229B* | 1 | 101.015 |
| *FKBP7* | 1 | 102 |
| *PLN* | 1 | 102 |
| *PLA2G5* | 1 | 103 |
| *CH25H* | 1 | 104 |
| *OGN* | 2 | 222 |
| *KRT2* | 5 | 545.106 |
| *SCARNA1* | 1 | 115 |
| *PRKAR2B* | 30 | 3679.93 |
| *COMMD3-BMI1* | 2.77719 | 410.728 |
| *CD69* | 1 | 151.518 |
| *VSIG4* | 1 | 182 |
| *FGF7* | 2 | 329.521 |
| *RTEL1-TNFRSF6B* | 0.133625 | 27.2748 |
| *HRASLS5* | 4 | 824.418 |
| *C8orf44-SGK3* | 0.163743 | 41.0779 |
| *LYVE1* | 1 | 356.514 |
| *C20orf24* | 0.0803608 | 38.0704 |
| *CCL21* | 1 | 436 |
| *MME* | 2 | 1236 |
| *ANKRD20A3_dup2* | 0.00794651 | 4.99385 |
| *FAM72A* | 0.00572457 | 7.99987 |
| *GTF2H2C* | 0.00404863 | 13.855 |
| *COX20* | 0.0164124 | 67.1649 |
| *RPL17-C18orf32* | 0.00308888 | 14.5601 |
| *FAM133B* | 0.00480433 | 22.9479 |
| *BLOC1S5-TXNDC5* | 0.0636358 | 335.086 |
| *SYNE1-AS1* | 0.0203846 | 112.177 |
| *MSTO2P_dup1* | 0.00264817 | 14.6958 |
| *RGPD1_dup1* | 0.0017397 | 11.5836 |
| *MEF2BNB-MEF2B* | 0.00091512 | 20.0425 |
| *PDF* | 0.00049202 | 11.1008 |
| *TCEB3CL_dup2* | 0.00043606 | 9.97243 |
| *CCDC169-SOHLH2* | 0.00037486 | 9.67815 |
| *SCO2* | 0.00013221 | 6.6897 |
| *P2RY11* | 5.78E-05 | 4.46691 |
| *LOC100506076_dup2* | 3.22E-05 | 6.0115 |
| *TMEM183A* | 8.83E-05 | 29.2667 |
| *ZNF559-ZNF177* | 4.17E-05 | 22.4618 |
| *HSPE1-MOB4* | 7.50E-06 | 4.65745 |
| *RGPD1_dup2* | 4.19E-05 | 26.9293 |
| *ZNF321P* | 1.38E-05 | 23.944 |
| *RNASEK-C17orf49* | 2.59E-06 | 7.11781 |
| *ELMO1-AS1* | 1.15E-06 | 7.14787 |
| *FAM27A_dup1* | 1.13E-06 | 10 |
| *TICAM2* | 1.57E-06 | 18.9268 |
| *HYI* | 3.91E-07 | 37.5058 |
| *KLRK1* | 2.23E-09 | 25.9999 |
| *CD302* | 1.72E-08 | 411.656 |
